# A Systematic Review of the Therapeutic Role of Gastric Pacemakers in Adults With Gastroparesis

**DOI:** 10.7759/cureus.18152

**Published:** 2021-09-21

**Authors:** Medha Rajamanuri, Sai Mahitha Mannava, Jayksh Chhabra, Guruprasad Vasant Karwarker, Meher Chahal, Anand Reddy Maligireddy, Eiman Dai, Michael Alfonso

**Affiliations:** 1 Internal Medicine, California Institute of Behavioral Neurosciences & Psychology, Fairfield, USA; 2 Pediatrics, California Institute of Behavioral Neurosciences & Psychology, Fairfield, USA; 3 Psychiatry, California Institute of Behavioral Neurosciences & Psychology, Fairfield, USA; 4 Medicine, California Institute of Behavioral Neurosciences and Psychology, Fairfield, USA

**Keywords:** gastric stasis, gastroparesis, gastric electrical stimulation, nausea, vomiting, gastroparesis, abdominal bloating, gastric neuromodulation

## Abstract

Gastroparesis or gastric stasis is the delayed transit of the ingested contents through the stomach in the absence of mechanical obstruction. It can have multiple etiologies, most commonly idiopathic (ID) and diabetic (DM). Gastroparesis can cause significant distress to patients as it leads to symptoms like intractable nausea and vomiting, weight loss, abdominal bloating, early satiety, etc. The pathogenesis is mainly thought to be due to the dysfunction of the gastric pacemaker cells, i.e., interstitial cells of Cajal (ICC), and their interaction with the other gastric motor function regulatory components. There are several proposed treatment options for gastroparesis. Despite that, most patients remain refractory to medical treatment and require additional interventions for symptomatic relief. One such intervention is gastric electrical stimulation or gastric pacemaker, which aids in improving gastric motility. We have searched PubMed, PubMed Central (PMC), Medline, Science Direct, and Google Scholar for articles pertaining to the use of gastric electrical stimulation in gastroparesis published in the last 10 years. The keywords used include "gastroparesis", "gastric stasis", "gastric pacemaker'', "gastric electrical stimulation", "nausea", "vomiting", "abdominal bloating", "gastric neuromodulation". We have finally included twelve studies that were the most relevant to our research question and met the quality assessment criteria. Exclusion criteria consisted of pediatric population studies, studies conducted on animals, books, and grey literature. Overall, these twelve studies helped evaluate the impact of gastric pacemakers on symptoms of gastroparesis like nausea, vomiting, weight loss, abdominal bloating, and quality of life. We found that most studies favored gastric pacemakers, improving the incidence of nausea and vomiting in patients with gastroparesis. There was a marked improvement in the BMI as well. On the other hand, most open-labeled studies showed improved quality of life and Gastroparesis Cardinal Symptom Index (GCSI) scores, while randomized controlled trials and meta-analyses did not reflect the same result. In addition, some other parameters improved with gastric pacemakers, Inflammatory markers, insulin levels (especially in diabetics), and the number of hospitalizations. In conclusion, gastric pacemaker is a potential treatment option for patients with medically refractory gastroparesis. As noted from the results of our study, nausea/vomiting, weight loss, and overall GCSI scores have shown marked improvement with gastric electrical stimulation (GES). Nevertheless, more extensive research is needed to understand better the full extent of this device’s use as a viable treatment option for patients suffering from gastroparesis.

## Introduction and background

Gastroparesis (GP) is chronic and often disabling neuromuscular disorder of the upper gastrointestinal tract [1]. It is characterized by impaired gastric motility in the absence of mechanical obstruction of the stomach [2]. Symptoms most commonly include nausea, vomiting, early satiety, postprandial fullness, upper abdominal pain, bloating, and weight loss [3]. Aetiologies of gastroparesis can be idiopathic, diabetic, iatrogenic, post-surgical, or post-viral [4]. In the United States, the incidence of hospitalizations related to gastroparesis has increased substantially since 1995 and particularly after 2000. In one only community-based study, the age-adjusted prevalence of idiopathic gastroparesis per 100,000 persons was higher in women than men. In another study about gastroparesis in diabetes mellitus (DM), the average cumulative incidence of symptoms and delayed gastric emptying over 10 years was higher (up to 5%) in type 1 DM than in type 2 DM (1%) [5].

The underlying pathophysiological mechanisms are complex. Some of the involved mechanisms include abnormal gastric motility (accommodation, emptying), autonomic dysfunction, visceral hypersensitivity, low-grade mucosal inflammation, and cellular changes in enteric nerves, muscle, or interstitial cells of Cajal [6,7]. The current treatment strategies primarily include symptomatic management and dietary measures, fluid therapy, prokinetic drugs, gastric electrical stimulation, and endoscopic or surgical intervention [8].

**Figure 1:**
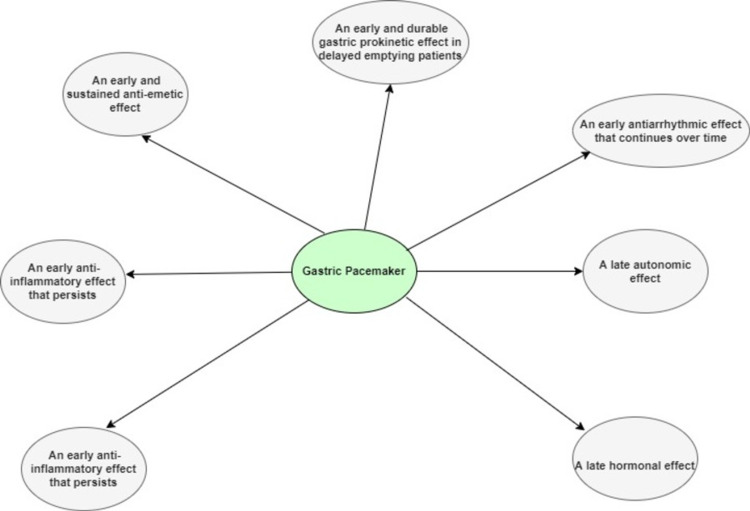
The possible beneficial effects of a gastric pacemaker

Gastric pacemaker therapy or gastric electrical stimulation (GES) has been proposed as an alternative effective treatment option for patients with gastroparesis whose symptoms persist despite medical therapy [9]. Recent studies suggest that electrical stimulation improves symptoms and physiology with (a) an early and sustained anti-emetic effect; (b) an early and durable gastric prokinetic effect in delayed emptying patients; (c) an early antiarrhythmic effect that continues over time; (d) a late autonomic effect; (e) a late hormonal effect; (f) an early anti-inflammatory effect that persists; and (g) an early and sustained improvement in health-related quality of life [10]. (Figure 1)

Gastric electrical stimulation offers a variety of potential benefits, including synchronization of intrinsic gastric electrical activity, evoking propagating contractions, and alleviating symptomatology in individuals with gastroparesis, depending on stimulus settings and stimulation locations. Extra-intestinal effects of gastric stimulation parameters include alterations in systemic hormonal and autonomic neural activity, as well as modification of afferent nerve pathways projecting to the central nervous system, which might be significant mechanisms of action [11].

Currently, pathophysiological research continues to focus on the cause of disordered motility and visceral hypersensitivity in the pathogenesis of GP symptoms. Studies show that patients with gastroparesis had different clinical outcomes after GES therapy based on underlying etiology [12].

A better understanding of the targeted neural circuits and their physiological and pathophysiological roles, as well as improving stimulation regimens and discovering which patients benefit the most from this therapy, could improve the clinical success of the GES [13]. In this review, we aim to understand some of the alterations leading to disorders such as gastroparesis and the effectiveness of gastric electrical stimulation as a potential therapeutic option for relieving the symptoms of gastroparesis in various patient populations.

## Review

Methods

The databases used for this systematic review include PubMed, PMC, Medline, Science Direct, and Google Scholar. The keywords that were searched are “Gastroparesis”, “Gastric stasis”, “Gastric pacemaker”, “Gastric electrical stimulation”, “Nausea”, “Vomiting”, “abdominal bloating", "Gastric neuromodulation”.

Data Extraction

**Figure 2:**
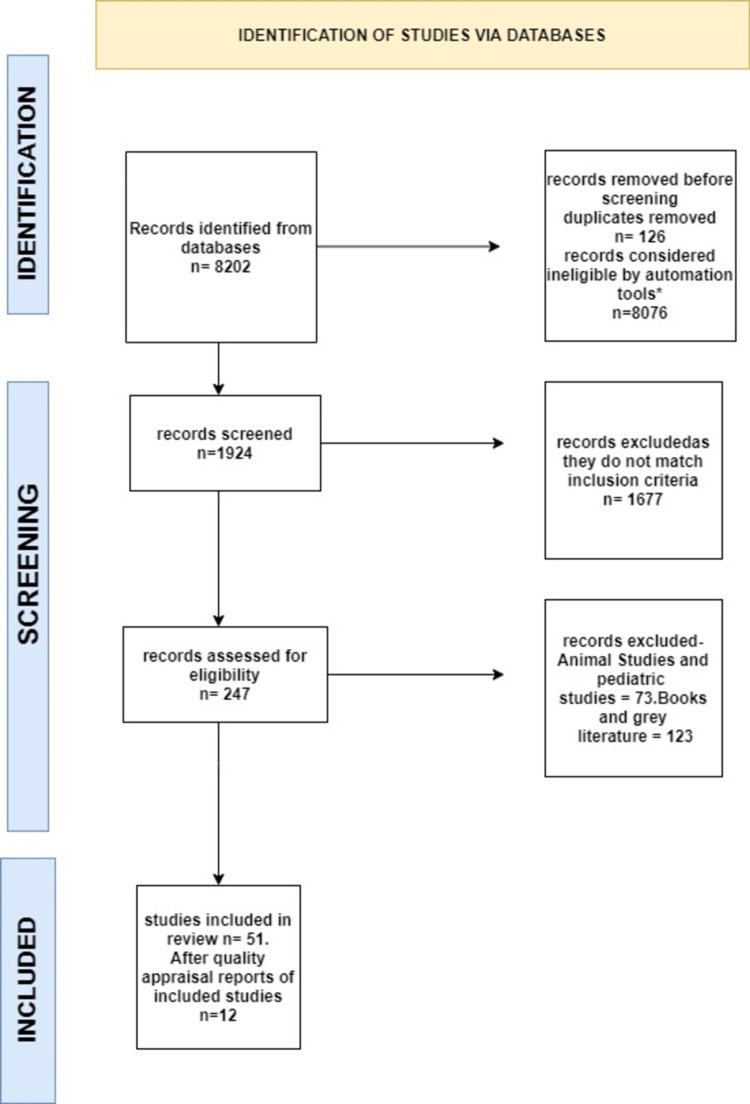
Data extraction process using the PRISMA flow diagram *Results from the last 10 years.

Two reviewers went through a total of 1924 articles and identified them to be relevant to either gastroparesis and its symptoms or the gastric electrical stimulation therapy in all the databases after exclusion of duplicates. Two investigators had screened the titles of the publications identified independently. After careful screening of titles, if all the inclusion criteria were met and the article suited the research question, the full text was obtained. Of all these articles, 124 met initial inclusion and exclusion criteria, 41 articles were found to be most relevant, and were available with abstracts. Out of which, we included 12 studies with full text in the results. The data extraction has been presented in the form of Preferred Reporting Items for Systematic reviews and Meta-Analyses (PRISMA) flow chart below (Figure 2) [14]. The review has been carefully drafted following the PRISMA guidelines for systematic review [14].

Inclusion Criteria

The following inclusion criteria were used: studies published in the last 10 years; studies conducted on humans and published in English; studies including data on adults with medically refractory gastroparesis that required gastric electrical stimulation therapy; and peer review papers. We included review articles, observational studies, and clinical trials.

Exclusion Criteria

The following exclusion criteria were applied: studies involving the pediatric population, studies conducted on animals, books, and grey literature.

Quality Appraisal

The quality of all 12 studies was assessed based on different tools for each type of study: 1) Cochrane risk bias assessment tool for randomized clinical trials; 2) Newcastle Ottawa scale for observational studies; 3) AMSTAR2 was used to assess the quality of systematic reviews; 4) JB checklist for case series; 5) Sanra checklist to assess the quality of reviews.

Results

Of all the relevant articles found, 12 articles have been analyzed, and the results are presented below. These studies helped evaluate gastric pacemaker's impact on symptoms of gastroparesis like nausea, vomiting, weight loss, abdominal bloating, and quality of life. Table 1 presents the results of various clinical trials, other clinical studies, systematic reviews, and meta-analyses published in the last 10 years.

**Table 1:** Description of the purpose, authors, results, and outcomes of the studies included in this review. GES - gastric electrical stimulation; TSS - total symptom severity; GCSI - gastroparesis cardinal symptom index; Padj - adjusted p-value; DM - diabetes mellitus, ID- idiopathic, PS - post-surgical, PSG - post-surgical gastroparesis

Author/year	Purpose	Type of study	Subjects	Results	Outcomes
1. Hedjoudje et al. (2020) [15]	At 10 years, the efficacy of stomach electrical stimulation in the treatment of persistent nausea and vomiting.	A retrospective single-center study	50	Comparing means pre-implantation vs 10-year follow-up. Improvement in early satiety (3.05 vs 1.76, p<0.001); nausea (2.46 vs 1.35, p=0.001); vomiting (3.35 vs 1.45, p<0.001); BMI (23.40 vs 26.46, p= 0.048).	A significant relief of early satiety, nausea, vomiting was noted, along with improvement of BMI.
2. Kim et al. (2020) [16]	Does the etiology of gastroparesis influence the clinical results of gastroparesis therapy with gastric electrical stimulation?	A retrospective cohort	183 patients subgroups- DM=91 ID=76 PS=16	DM patients saw a greater incidence of weight gain > 4 kg, compared to PS and ID patients (67.6% vs 8.1% vs. 24.3%, respectively, p < 0.05).	Significant weight gain was observed with GES in all patients, but it was significantly more in diabetics
3. Ducrotte et al. (2020) [17]	In a randomized controlled trial, to test if gastric electrical stimulation improved refractory vomiting.	A large randomized, multicenter, double-blind trial with crossover	172	In diabetic and nondiabetic individuals, vomiting ratings were greater in the group with the device on (median score, 2) versus the control group (median score, 1; P = .001). In individuals with delayed (P = 0.01) or normal stomach emptying (P = 0.05), vomiting scores rose considerably when the device was turned on.	It was found that GES reduced the frequency of refractory vomiting, improving vomiting scores whereas improved quality of life and accelerated gastric emptying were not seen.
4.Shine et al. (2019) [18]	To assess the significance of gastric electrical stimulation (GES) in gastroparesis treatment.	Review article	34 articles on the role of GES.	Baseline after both temporary and permanent GES at 5–7 days and 6 months, respectively were measured. GCSI for nausea reduced from a baseline level of 3.5 to 1.7 and 2.6. GCSI for vomiting reduced from the baseline of 2.4 to 0.6 and 1.8. Total liquid emptying delay significantly decreased, from 94% at baseline to 52% and 58%. Total solid emptying decreased from 152% to 105% and 100%.	A significant improvement was seen in nausea and vomiting scores. There is an overall improvement in liquid and solid emptying from the stomach. Inflammatory markers have also shown a significant reduction after GES therapy.
5. Shada et al. (2018) [19]	A multi-institutional study of gastric electrical stimulation for medically resistant gastroparesis: Wisconsin's Enterra therapy experience.	Observational study	119 (64 diabetic and 55 idiopathic)	GCSI scores improved, and prokinetic and narcotic medication use decreased significantly at ≥1 year. Satisfaction scores were high.	Improvement in GCSI scores.
6. Corvinus et al. (2018) [20]	A pilot research to predict the outcome of electronic gastric stimulation with the Enterra TM system using minimally invasive temporary gastric stimulation.	Case series	6	Baseline and postoperative gastroparesis cardinal symptom index (GCSI), a validated index for gastroparesis therapy, was assessed. Response to EGS was defined as a 50% decrease of baseline GCSI. Four of six patients responded to temporary EGS. Three of four responders underwent permanent implantation. One non-responder received a permanent Enterra™ at another institution. After a median follow up time of nine months GCSI remained low in the responder group.	Improvement in GCSI scores post gastric pacemaker transplant.
7. Laine et al. (2018) [21]	This is a retrospective multi-centric research on the results of high-frequency GES for the treatment of severe, medically refractory gastroparesis in Finland.	A retrospective multi-center cohort comprising of all patients who had been implanted with gastric electric stimulator for severe, medically refractory gastroparesis.	13	Eleven patients (79%) gained a median of 5.1 kg in weight (P < 0.01), and symptoms were relieved significantly in eight and partially in three patients (79%).	Symptomatic improvement, including weight gain, was noted.
8. Levinthal et al. (2017) [22]	Review gastric electrical stimulation for gastroparesis between 1990 - 2014.	Systematic review and meta-analysis	5 controlled trials and 16 open-labeled studies.	Total symptom severity (TSS) scores did not differ between these periods in the controlled trials (0.17 [95% confidence interval: −0.06 to 0.4]; P = 0.15). However, sixteen open-label studies of GES showed a significant total symptom severity score decrease (2.68 [2.04–3.32];P < 0.001). Some other treatment modalities similarly improved TSS by 1.97 [1.5–2.44],1.52 [0.9–2.15], and 2.32 [1.56–3.06] for medical therapy (MED), placebo arms (PLA), and botulinum toxin (BTx) respectively.	The TSS scores were better post GES therapy in open labeled studies but did not show significant differences in randomized trials.
9. Lahr et al. (2013) [23]	The impact of GES on abdominal pain.	Clinical trial	95	At baseline, 68 patients reported severe pain. In these patients, mean pain scores decreased with temporary GES from 3.62 to 1.29 (P < 0.001) and pain that is not severe from 1.26 to 0.67 (P = 0.01). Upon using permanent GES, severe mean pain scores were reduced to 2.30 (P < 0.001); pain that is not severe showed a non-significant increase to 1.60 (P = 0.221). Mean follow-up was 275 days.	Patients with severe abdominal pain showed improvement with both temporary and permanent gastric pacemaker placement. Whereas patients with non-severe pain only showed significant improvement with a temporary pacemaker.
10. McCallum et al. (2020) [24]	GES with Enterra therapy improving gastroparesis symptoms.	Randomized controlled trial	32	The initial unblinded ON period indicated decreased weekly vomiting frequency (WVF) from baseline (61.2%, P < 0.001). After 1 year of therapy, WVF was still low (median reduction = 87%, P < 0.001),along with symptomatic improvement in GP symptoms, gastric emptying and duration of hospital stay (P < 0.05).	Initially, there was reduced vomiting during the ON phase of GES. Analysis after 3 months did not show a reduction in vomiting in the ON as compared to the OFF period (iii), After a year of ON stimulation, vomiting scores were consistently low along with lower hospitalizations.
11. Abell et al. (2011) [25]	To study the effects of a temporary gastric pacemaker on gastroparesis symptoms.	Randomized controlled trial	58 patients (ID- 38; DM-13, PS- 7)	Results were pooled from 2 separate sessions of GES therapy, this indicated that there is no significant decrease in daily average vomiting scores. 0.12 (-0.26 to 0.03; P = .116).	There was no significant overall effect of GES on vomiting scores but the differences favored stimulation.
12. Chu et al. (2012) [26]	Usage of high-frequency GES for treatment of gastroparesis.	Review	10 studies (n=601)	Significant improvement was noted in both TSS (P < 0.00001) as well as retention of gastric contents at two hours (P = 0.003) and four hours (P < 0.0001) in diabetic gastroparesis patients (DG), while gastric retention at two hours (P = 0.18) in patients with idiopathic Gastroparesis (IG), and retention of gastric contents at four hours (P = 0.23) in post-surgical Gastroparesis (PSG) patients was insignificant	Response to GES is more pronounced in diabetic gastroparesis. ID and PSG have shown a weaker response to this therapy.

Discussion

**Figure 3:**
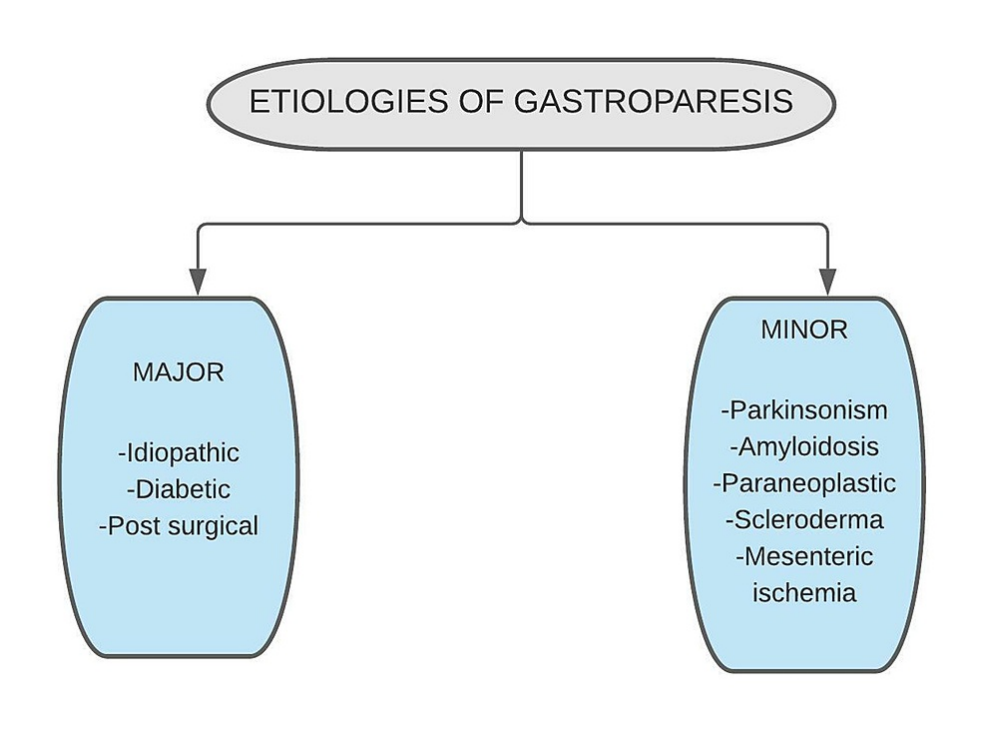
Etiologies of gastroparesis

Gastroparesis is a clinical illness characterized by symptoms suggestive of altered digestive function of the proximal gastrointestinal tract and objective evidence of abnormally prolonged retention of stomach contents in the absence of apparent mechanical obstruction [27]. Most gastroparesis cases can be divided into three categories: idiopathic gastroparesis (ID, 36%), diabetic gastroparesis (DG, 29%), and postsurgical gastroparesis (PSG, 13%). Idiopathic Gastroparesis refers to a symptomatic patient with delayed stomach emptying with no identifiable fundamental underlying problem. ID is the most common form of gastroparesis. Most patients with ID are typically young or middle-aged women [28]. One significant cohort of ID is post-viral; these patients present with rapid onset of GP symptoms after a phase of viral prodrome. Typically, patients have a rapid onset of GP symptoms with intractable nausea and vomiting, and they improve over a year. In comparison, the most identified cause of gastroparesis is diabetes [29]. In the NIH consortium cohort, type 1 DM patients had more delayed gastric emptying [4]. The 10-year incidence of gastroparesis has been recorded as 5.2 % in type 1 diabetes, 1 % in type 2 diabetes, and 0.2 % in non-diabetic controls in a US community [28]. Other less common causes of gastroparesis are Parkinsonism, amyloidosis, paraneoplastic, scleroderma, and mesenteric ischemia [29] (shown in Figure 3).

Clinical implications

Nausea, vomiting, early satiety, postprandial fullness, bloating, belching, and upper abdominal discomfort are all signs of Gastroparesis, which may overlap with symptoms of functional dyspepsia and rapid gastric emptying. The Gastroparesis Symptom Severity Scale is one of several symptom severity ratings used as patient-reported symptom assessments in gastroparesis, including the Gastroparesis Symptom Index (GCSI, shown in Table 2), which is based on the comprehensive Patients Assessment of Upper Gastrointestinal Disorders-Symptoms and the revised GCSI-Daily Diary (GCSI-DD). These scales have been used in clinical trials to assess the effects of treatment in clinical studies of gastroparesis.

**Table 2:** GCSI scoring This table provides a description of the Gastroparesis Cardinal Symptom Index (GCSI) and its components. Score each of nine symptom criteria on 0 (none) to 5 (very severe).

Nausea/vomiting	Postprandial satiety	Bloating
1. Nausea	4. Stomach fullness	8. Stomach visibly larger
2. Retching	5. Not able to finish a normal-sized meal	9. Bloating
3. Vomiting	6. Feeling excessively full after meals	
	7. Loss of appetite	

Symptom profiling of patients recruited in the Gastroparesis Clinical Research Consortium (GpCRC) revealed that 50-60% of gastroparesis patients felt severe early satiety and postprandial fullness, with DG and ID having similar severity levels. Nausea was noted by 95% of the patients (predominant symptom in 29 percent). It was linked to meals in three-quarters of the patients, and it lasted most of the day in more than 40% of them. Nausea/vomiting sub-score of the Patient's Assessment of Upper Gastrointestinal Disorders-Symptoms (PAGI-SYM) was more significant in DG patients, with vomiting lasting several hours or most of the day in more than half of the DG patients compared to 24% in ID. Patients with DG have also reported vomiting before eating in the morning. Forty percent of gastroparetics experienced severe bloating (GCSI 4 of 5), and it was linked to the female sex, overweight status, changed bowel function, and probiotic use [27].

Physiopathogenesis

**Figure 4:**
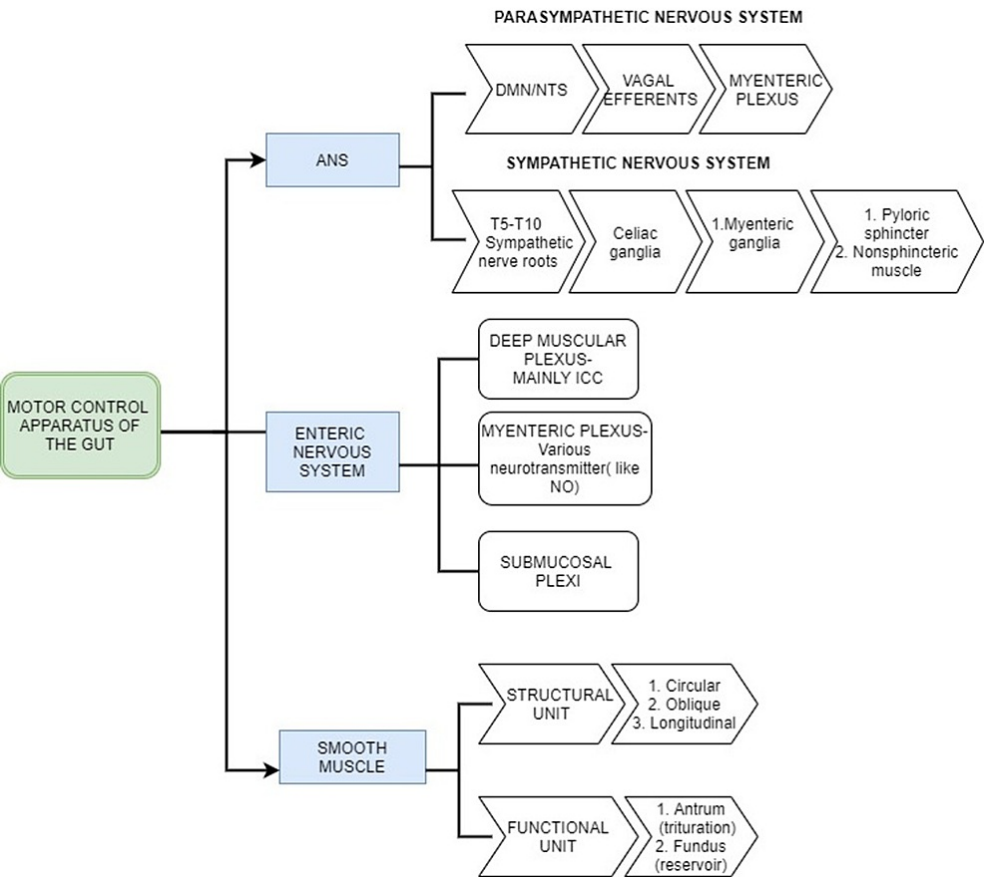
Gastric motor control apparatus This figure represents the motor control apparatus of the gut and its components.

A succession of complex and well-coordinated muscular and secretory processes culminates in emptying a meal from the stomach into the small bowel. The fundus, antrum, and pylorus are all implicated in gastroesophageal (GE) from a neuromuscular viewpoint (components of gastric motility have been demonstrated in Figure 4). Swallowing causes the gastric fundus to relax actively, allowing it to take vast amounts of food without causing intragastric pressure to rise. Following that, a continuous increase in fundal tone drives stomach contents toward a fast-closing pylorus, where digestible solids are mashed along with gastric secretions and bounced back into the proximal stomach. The interstitial cells of Cajal (ICC) that are situated in the upper portion of the greater curvature and generate a slow-wave basal electrical rhythm (pacesetter potential) with a frequency of three depolarizations per minute control the maximal frequency of antral contractions. This process continues until all digestible solids are reduced to particles of 2 mm or less, and tiny quantities of fluids and homogenized food (chyme) escape the stomach shortly before pyloric contractions. Normal small bowel function is also required for GE, not only because antro-pyloro coordination is needed to empty the stomach, but also because neuro-endocrine inhibitory signals arise from both the proximal and distal small bowels, based on the composition of the chyme, to modulate emptying rates, ensuring that delivery to the absorbent mucosa matches liver and pancreas secretory activities [27].

The connection between delayed stomach emptying and symptom pattern in Gastroparesis and the distinction between functional dyspepsia and gastroparesis is still a source of debate and ambiguity. The function of pyloric resistance and duodenal motility in developing symptoms has been studied in pathophysiological investigations. Glycemic management did not influence short-term changes in stomach emptying rate in diabetic patients with type 2 diabetes. Still, it was a key risk factor for the long-term development of gastroparesis in type 1 diabetes patients. Diabetic gastroparesis is characterized by a loss of ICCs at the cellular level, which is negatively associated with the amount of CD206+ macrophages, which are considered to protect ICCs. The SIP syncytium (Smooth muscle cells are electrically coupled to ICC and PDGFRα(+) cells, forming an integrated unit called the SIP syncytium), rather than the ICC alone, is now recognized as the pace making unit in the pathophysiology of GP, and GP may be part of a pan-enteric autoimmune and/or autonomic disease with macrophage imbalance [30,31].

Currently available treatment modalities

**Figure 5:**
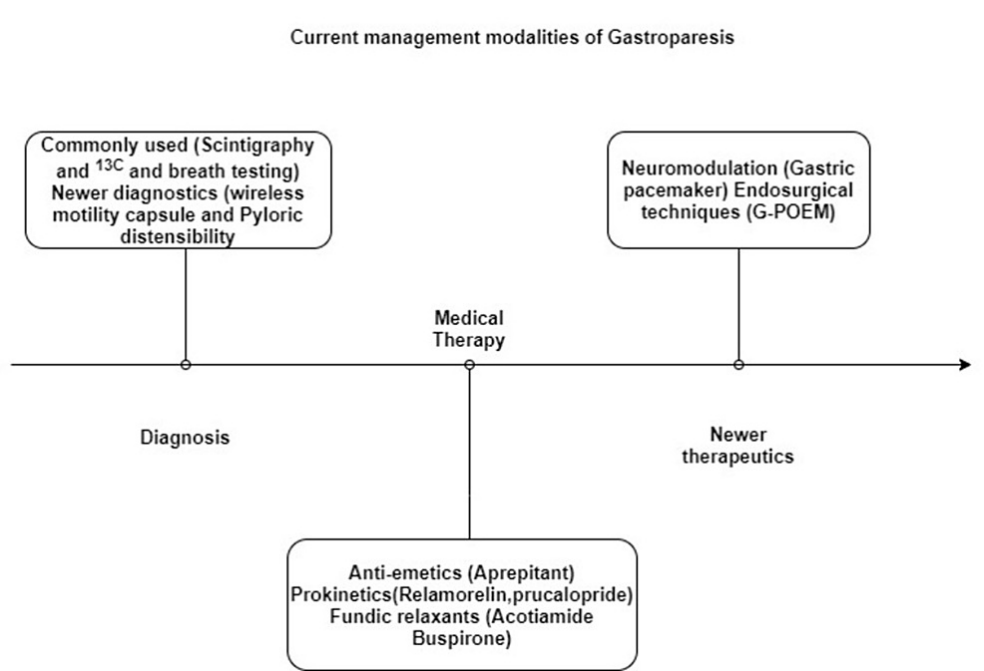
A timeline chart of current management strategies of gastroparesis G-POEM - gastric peroral endoscopic myotomy

As discussed in the previous section, there are several complex mechanisms in the gut that cause gastroparesis. Scintigraphy and 13 C breath testing are the best assessment tools for GE. Newer diagnostic modalities like wireless motility capsules and pyloric distensibility (EndoFLIP) are aiding in better characterization of this disease. The only effective medical treatment for gastroparesis has been the use of metoclopramide. Antiemetics (aprepitant), prokinetics (relamorelin, prucalopride), and fundic relaxants are some of the more recent therapeutic options (acotiamide, buspirone). In addition to pharmaceutical compounds in the pipeline, neuromodulation and endosurgical techniques, such as gastric peroral endoscopic myotomy (G-POEM), may help address refractory. Endoscopic pyloromyotomy appears to be effective in the short term, especially for nausea and vomiting, but further study is needed to accurately identify the subset of gastroparesis patients who have pyloric dysfunction, and long-term results must be assessed [15,4] (Figure 5).

Gastric pacemaker/ gastric electrical stimulator

The FDA has approved the use of gastric electrical stimulators (GES) for drug-resistant IG and DG. The device is placed directly into the stomach. GES has been recommended as a treatment option for people who are resistant to medicines.

**Figure 6:**
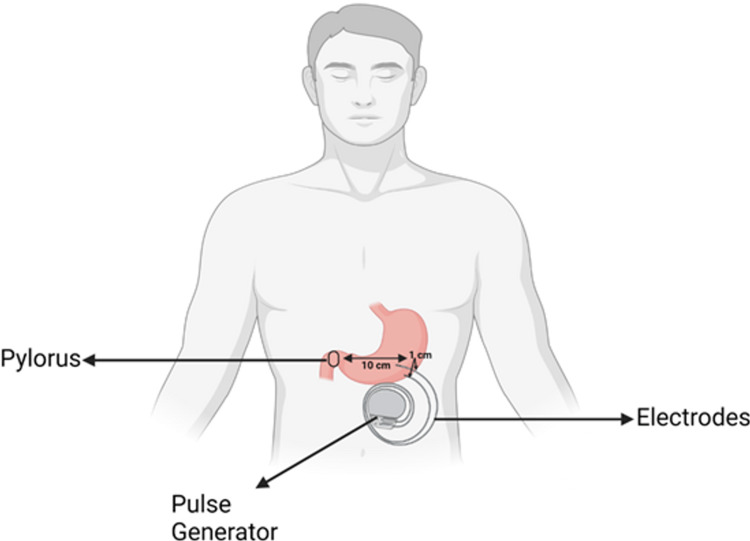
Positioning of gastric pacemaker in relation to the greater curvature and pylorus of the stomach

The device is made up of electrodes surgically implanted in the stomach's anterior wall and coupled to a pulse generator [29]. The Enterra stomach electrical stimulator is implanted by surgery. A laparotomy or a less invasive laparoscopy may be used by the surgeon. The complete device is made up of two leads, a pulse generator, and a programming system, all produced by Medtronic (Medtronic, Dublin, Ireland). At a distance of ten centimeters proximal to the pylorus, the two neuromuscular leads are inserted 1 cm apart within the muscularis propria of the stomach's greater curvature. The stimulation settings for the Medtronic Model 4351 pulse generator are as follows: amplitude: 5 mA, pulse width: 330 s, and cycle: 12 cpm (on time: 0.1 s-14 Hz; off time: 5.0 s). The pulse generator is generally located in the upper right or left quadrant of the abdomen wall. Different stimulation parameters can be programmed using an external device. High-frequency and low-energy settings are used in Enterra treatment stimulation. The battery lasts about 5 to 10 years in most cases. If a battery must be replaced, the electrodes do not have to be replaced [32] (Figure 6).

Gastrointestinal symptoms

Nausea and vomiting are the most frequent and bothersome symptoms experienced by patients with gastroparesis. The gastric pacemaker has been shown to help alleviate these symptoms, as described in the studies in our review [18]. A study conducted between 1998 to 2009 on 50 patients revealed that there was an improvement in nausea (2.46 vs. 1.35, P =.001) and vomiting (3.35 vs. 1.49, P<0.001) [15]. In another study conducted on 13 patients by Laine et al. showed that before implantation, 13 had severe nausea, 11 had severe vomiting. After the pacemaker implantation procedure, eight patients reported significant relief of these symptoms, and three said partial replacement. One patient did not report any improvement in nausea/vomiting but had substantial weight gain [21].

In a randomized crossover study conducted by Ducrotte et al., GES reduced the frequency of refractory vomiting in a total of 172 patients with and without diabetes [17]. GCSI scale was used to analyze the improvement of nausea and vomiting scores, showing that nausea scores dropped from 3.5 at baseline to 1.7 and 2.6 and six weeks and six months, respectively. Similarly, vomiting scores had dropped from 2.4 to 0.6 and 1.8 as published in a review made by Shine et al. [18].

Another randomized controlled trial published by McCullum et al. presents reduced vomiting scores immediately after induction of GES therapy and after a year of continuous usage whereas, there was no improvement seen when analyzed at three months after the start of GES therapy [24]. In contrast, a randomized controlled trial comprising of 58 patients, conducted by Abell et al., demonstrated no significant reduction in vomiting scores with GES therapy [25].

Abdominal bloating, early satiety, and slow gastric emptying symptoms- Impact of GES on these symptoms was evaluated in a study published by Hedjoudje et al. In 2020 with a total of 50 patients who were implanted with Gastric pacemakers from January 1998 to December 2009 demonstrated that beyond ten years, there is an improvement in early satiety (3.05 vs. 1.76, p <0.001), bloating (2.51 in comparison to 1.70, P =.012) [15]. According to a review published by Shine et al. in 2019, compared to baseline gastric emptying delay, total liquid emptying delay significantly decreased, from 94% at baseline to 52% and 58%, using a temporary and permanent GES, respectively. In addition, baseline total solid emptying delay decreased from 152% to 105% and 100% at six days and six months [18]. About abdominal pain, in a clinical trial conducted by Lahr et al., patients suffering from severe abdominal pain had improved significantly with both temporary and permanent placement of gastric pacemakers [23].

Overall symptomatic improvement, weight loss, and quality of life

GCSI and total symptom severity (TSS) scores have been used to gauge the impact of GES on a patient's symptoms due to gastroparesis.

Wisconsin's Enterra therapy experience, a clinical study, has demonstrated that GCSI scores improved, and prokinetic and narcotic medication use decreased significantly at ≥ one year. Satisfaction scores were high with the help of gastric pacemakers in 119 patients with gastroparesis, of which 64 had idiopathic gastroparesis, and 45 had diabetic Gastroparesis [19].

In another study published by Corvinus et al. in 2018, Baseline and postoperative gastroparesis cardinal symptom index (GCSI), a validated index for GP therapy, was assessed. Response to electrical gastric stimulation (EGS) was defined as a 50% decrease of baseline GCSI. After a median follow-up time of 9 months, GCSI remained low in the responder group (four out of six patients had responded to EGS therapy) [20]

Levinthal et al. had published a systematic review and meta-analysis in 2017, which included articles from 1990-2014 on the effect of GES on total symptom severity score. Although total symptom severity scores did not differ in the five controlled trials (0.17 [95% confidence interval: −0.06 to 0.4]; P = 0.15), 16 open-label studies of GES showed a significantly lower TSS (2.68 [2.04-3.32]; Q = 39.0; P < 0.001). But the three studies showed significant differences in baseline TSS ratings (GES: 6.28 [6.28-7.42]; PLA: 4.59 [3.77-5.42]; MED: 4.76 [4.09-5.42]; BTx: 6.02 [5.3-6.74]; Q = 35.1; P b 0.001). Meta-analysis revealed that these baseline differences seemed to have impacted the results of the TSS ratings during treatment [22].

A review published by Chu et al. involving 601 subjects concluded that the beneficial effects of GES were seen more in patients with diabetic Gastroparesis than post-surgical and idiopathic etiologies [26].

A weight loss-A study was conducted with 183 patients who underwent GES from 2005 to 2015. Of the 183 patients with gastroparesis. 50% were diabetic, 42% idiopathic (ID), and 9% post-surgical (PS). The results demonstrate that DM patients saw a greater incidence of weight gain > 4 kg than PS and ID patients (67.6% vs. 8.1% vs. 24.3%, respectively, p < 0.05) [16].

A retrospective multi-center cohort study with 13 patients implanted with GES also supported the benefit of using GES for patients suffering from weight loss due to gastroparesis. According to this study, 11 patients (79%) gained a median of 5.1 kg in weight (P < 0.01) [21]. Another study with positive results is a retrospective single-center study with 50 participants showing improved BMI post gastric pacemaker implantation (23.40 vs. 26.46, p= 0.048) [15].

Quality of life- a large, multi-center, randomized, double-blind trial with a crossover consisting of 172 patients also did not show any significant difference in the quality of life among those with GES therapy [17]. On the other hand, Shine et al. published a study in 2019 showing a significant postoperative (implantation of gastric pacemaker) increase of 40.7% and 24.6% (p < 0.05) in quality-of-life scores [18].

Multiple studies have shown an improvement in quality of life (QOL) with the use of a gastric pacemaker, as measured by Medicare DRG-adapted tools like Short Form 36 (SF-36) or Investigator Derived Outcome Measurement System (IDIOMS). A substantial postoperative increase in quality-of-life scores of 40.7 percent and 24.6 percent (p 0.05) was observed in a long-term follow-up of 28 patients with GES that focused on nutritional elements of GP syndromes. Changes in overall IDIOMS scores with GES were larger in idiopathic patients (baseline:19.9, temp GES:12.8, perm GES:13.5) than diabetic patients (baseline:19.9, temp GES:12.8, perm GES:13.5) [18].

Other beneficial effects of GES

Inflammatory indicators improved with the long-term use of GES. At baseline, patients with gastroparesis symptoms exhibited increased levels of interleukin-6 (IL-6) (46.64 to 53.01 pg/mL) and tumor necrosis factor-alpha (TNF) (22.18 to 7.46 pg/mL) (normal Interleukin-6 10.1 pg/mL and TNF 7.1 pg/mL, respectively). After six days, IL-6 levels increased (141.74 to 133.04 pg/mL), but TNF levels began to decline (19.84 to 8.50 pg/mL). IL-6 (15.34 to 20.51 pg/mL) and TNF (6.58 to 2.58 pg/mL) levels were observed to have decreased to near normal ranges after six months of persistent GES (adjusted p-value (Padj) 0.001) [18].

All patients had late effects in metabolic hormones (insulin, glucagon, and amylin; Padj 0.001 for all). In comparison to individuals with idiopathic gastroparesis, diabetic patients had significantly greater blood serum glucagon (276 pg/mL (DM) vs. 189 pg/mL (ID), Padj = 0.03) and insulin (9373 pmol/L (DM) vs. 3652 pmol/L(ID), Padj = 0.03) after temporary GES. Many of these patients had aberrant hormone levels at the start of the study. Because of its effects on the endocrine and exocrine systems, GES could be used to treat specific causes of gastroparesis, whether diabetic or not [18].

Electrogastrogram frequencies in all patients at baseline, temporary (day six), permanent (month six) were 5.3 CPM, 4.6 CPM, and 4.4 CPM, respectively, and in patients with delayed stomach emptying (at baseline: 5.6 CPM, temporary GES: 4.7, permanent GES: 4.4). Individuals with diabetic gastroparesis (baseline: 4.9 CPM, temporary GES: 3.8, Padj = 0.02) experienced similar changes in mucosal electrogram (mEG) frequency, while patients with idiopathic gastroparesis (baseline: 4.6, temporary GES 4.1, Padj = 0.69) did not. Because gastroparesis can be caused by a lack of normal gastric electrical rhythm, GES' focused antiarrhythmic action may help restore baseline motility. This study found a significant reduction in hospital days following GES, with annualized median days in the hospital dropping from 2 at baseline to 0 at the end of a year (p = 0.006) [18]. Thus, fewer hospitalizations and shorter stays would result in fewer comorbidities, expenditures, and infections acquired in hospitals.

Limitations

**Figure 7:**
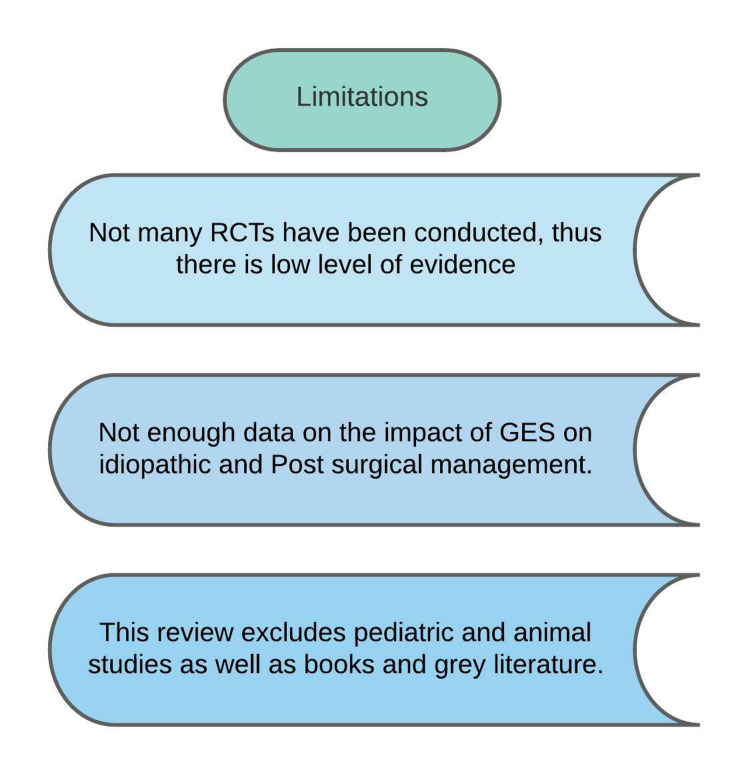
Illustration of the limitations of this study RCT - randomized controlled trial

Some of the studies included are open-labeled, which are inherently subject to bias. Therefore, further randomized controlled trials are needed to analyze the impact of gastric pacemakers in the improvement of symptoms in patients with gastroparesis. Some of the studies in this review suggest that symptomatic relief is more pronounced in patients with diabetic gastroparesis rather than Idiopathic and Post-surgical. More research is required to assess the use of gastric pacemakers in idiopathic and post-surgical gastroparesis. We have also only included relevant studies published in the last 10 years, out of which we have excluded the pediatric population, studies conducted on animals, books, and grey literature (Figure 7).

## Conclusions

The aim of this study is to see how effective stomach electrical stimulation is at treating gastroparesis symptoms like nausea, vomiting, abdominal bloating, weight loss, and overall quality of life. We found that gastric pacemakers have shown varying effects on each of these symptoms. In this review, most studies support the role of gastric pacemakers in reducing nausea and vomiting experienced by patients suffering from gastroparesis. Similarly, as evidenced by the studies reviewed, there was a significant weight gain noted with this therapy. Although most studies suggest a significant improvement in quality of life and GCSI scores, a few others suggest that there is no substantial change in the quality of life because of GES itself. However, the evidence supporting no difference in the quality of life seems stronger, as shown by the meta-analysis and randomized controlled trials vs. open-label trials that showed positive results for quality of life with gastric pacing. Some of the other parameters that have shown improvement after GES therapy include reduction in inflammatory markers, greater insulin levels (especially in diabetic patients), and reduction in hospitalizations.

We believe that, just as the cardiac pacemaker is extensively studied and used for managing cardiac dysrhythmias, the gastric pacemaker can be potentially used for managing GI dysrhythmias. However, the evidence supporting its use is currently limited; a lot more research is required in this field to harness the full ability of this device to improve the symptoms and, therefore, quality of life of patients with gastroparesis due to any aetiology.
